# Association of cervical carcinogenesis risk with HPV16 *E6* and *E7* variants in the Taizhou area, China

**DOI:** 10.1186/s12885-021-08531-y

**Published:** 2021-07-03

**Authors:** Mei-Zhen Dai, Yi Qiu, Xing-Hong Di, Wei-Wu Shi, Hui-Hui Xu

**Affiliations:** Medical Research Center, Taizhou Hospital of Zhejiang Province, Wenzhou Medical University, Linhai, Zhejiang, 317000 China

**Keywords:** HPV16, Genetic variants, *E6* gene, *E7* gene, Carcinogenesis, Cervical cancer

## Abstract

**Background:**

Human papillomavirus (HPV) type 16 accounts for a larger share of cervical cancer and has been a major health problem worldwide for decades. The progression of initial infection to cervical cancer has been linked to viral sequence properties; however, the role of HPV16 variants in the risk of cervical carcinogenesis, especially with longitudinal follow-up, is not fully understood in China.

**Methods:**

We aimed to investigate the genetic variability of HPV16 *E6* and *E7* oncogenes in isolates from cervical exfoliated cells. Between December 2012 and December 2014, a total of 310 single HPV16-positive samples were selected from women living in the Taizhou area, China. Sequences of all *E6* and *E7* oncogenes were analysed by PCR-sequencing assay. Detailed sequence comparison, genetic heterogeneity analyses and maximum-likelihood phylogenetic tree construction were performed with BioEdit Sequence Alignment Editor and MEGA X software. Data for cytology tests and histological diagnoses were obtained from our Taizhou Area Study with longitudinal follow-up for at least 5 years. The relationship between HPV16 variants and cervical carcinogenesis risk was analysed by the chi-square test or Fisher’s exact test.

**Results:**

In this study, we obtained 64 distinct variation patterns with the accession GenBank numbers MT681266-MT681329. Phylogenetic analysis revealed that 98.3% of HPV16 variants belong to lineage A, in which the A4 (Asian) sublineage was dominant (64.8%), followed by A2 (12.1%), A1 (11.4%), and A3 (10.0%). The A4 (Asian) sublineage had a higher risk of CIN2+ than the A1–3 (European) sublineages (OR = 2.69, 95% CI = 1.04–6.97, *P* < 0.05). Furthermore, nucleotide variation in HPV16 *E6* T178G is associated with the development of cervical cancer.

**Conclusion:**

These data could provide novel insights into the role of HPV16 variants in cervical carcinogenesis risk in China.

**Supplementary Information:**

The online version contains supplementary material available at 10.1186/s12885-021-08531-y.

## Background

Cervical cancer ranks as the fourth most common cancer in women worldwide, and approximately 90% of cervical cancer deaths occur in less developed countries [1]. Persistent human papillomavirus (HPV) infections are the major risk factor for cervical cancer, where nearly 100% of cervical cancer tissues contain HPV DNA [2]. Currently, more than 170 HPV types have been well identified, and approximately 40 of them can be easily transmitted in human mucosa or epithelium [3, 4]. Of the known oncogenic (or high-risk [HR]) HPV types, HPV16 is the most frequently (65%) involved in cervical cancer worldwide [5, 6]. Our previous epidemiological studies showed that the HPV16 type, with an infection rate of 3.1%, was less common in the Chinese general population but was found most frequently in patients with cervical intraepithelial neoplasia 2 (CIN2), CIN3 and cervical cancer (31.6, 47.9 and 60.6%, respectively) [7, 8]. Many lines of evidence have indicated that the genetic variability of the HPV16 genome contributes to HPV-driven cervical carcinogenesis [9, 10].

HPV16 genomes are approximately 8000 bp and composed of eight to nine open reading frames (ORFs) (*E1, E2, E4, E5, E6, E7, L1,* and *L2*) and a noncoding long control region (*LCR*) [9, 11]. E6 and E7 are the major oncoproteins that can rapidly degrade the tumour suppressor protein p53 and downregulate Rb products, resulting in cellular transformation and cervical carcinogenesis [12]. Based on genomic analysis, HPV16 genetic variants can be divided into four main phylogenetic lineages: A (sublineages A1–4), B (sublineages B1–4), C (sublineages C1–4) and D (sublineages D1–4) [13, 14]. The ethnicity of the sublineages is as follows: A1–3 (traditionally classified as European), A4 (Asian), B1–4 (African-1), C1–4 (African-2), D1 (North American), D2 and D3 (Asian-American), and D4 [15, 16]. The variants of HPV differ in carcinogenicity, geographical distribution, and ethnic group [16, 17]. In the present study, we investigated the genetic variants in HPV16 *E6* and *E7* oncoprotein-encoding genes and their involvement in cervical carcinogenesis in the Taizhou area, Southeast China.

## Methods

### Subject recruitment and HPV genotyping

Cervical exfoliated cell specimens were collected from Chinese patients who underwent cervical cancer screening at our gynaecological clinic from December 2012 to December 2014. HPV genotyping was performed using the GP5+/bioGP6 + −PCR/MPG assay for 27 genotypes using standard procedures (CFDA Certified No. (2017): 3404697) as previously reported [8]. The inclusion criteria were single HPV16-infected patients with no pregnancy and no history of total uterus or cervix resection. Total cellular DNA was extracted using the DNeasy Blood & Tissue Kit (QIAGEN) and stored at − 20 °C.

### PCR amplification and sequencing

For analysis of HPV16 variants, single HPV16-positive specimens were selected for this study. The HPV16 *E6* and *E7* genes were amplified by using specific primers for E6 and E7, 16E6E7_F 5′-ACTAAGGGCGTA ACCGAAAT-3′ and 16E6E7_R 5′-TGCAGTAAACAACGCAT-3′, which were designed using Primer Premier 5 according to the prototype HPV16 reference sequence (GenBank accession number K02718). PCR conditions consisted of 40 repeated cycles of 30 s denaturation at 94 °C, 45 s annealing at 57 °C, 45 s elongation at 72 °C, and a 7 min final incubation step at 72 °C (Thermo Hybaid, USA). PCR products had an amplicon size of 1061 bp (nucleotide sites [nt] 23–1083, including *E6* gene nt83–559 and *E7* gene nt562–858).

Subsequently, PCR products were purified and sequenced on the ABI 3730xl DNA Genetic Analyzer at BGI (Shanghai, China). To avoid PCR or sequencing errors, all the data were confirmed at least twice by repeating the PCR and sequencing reactions.

### Phylogenetic tree analysis

K02718 was used as the standard for alignment and nucleotide site numbering in this study. The *E6* and *E7* variants in the HPV16 sequences were aligned using the ClustalW tool applied by BioEdit Sequence Alignment Editor. Phylogenetic construction was carried out using MEGA X software using the maximum-likelihood statistical method [18]. To construct the phylogenetic branches, *E6* and *E7* in the HPV16 sequences were downloaded from GenBank NCBI and included K02718 (A1), AF536179 (A2), HQ644236 (A3), AF534061 (A4), KU053908 (B1), HQ644298 (B2), KU053915 (B3), KU053914 (B4), KU053917 (C1), HQ644244 (C2), KU053920 (C3), KU053925 (C4), HQ644257 (D1), AY686579 (D2), AF402678 (D3), and KU053931 (D4) [13, 15, 19].

### Treatment and follow-up

Patients with HPV16 infection were recommended for colposcopy examination immediately, according to the 2012 guidelines of cervical cancer screening [20]. Directed biopsy of suspected lesions was performed using cervical biopsy forceps. The biopsy tissues were placed in a 10% formalin solution for histological examination. Histological diagnoses were performed by pathologists and classified as normal, CIN1, CIN2, CIN3 or invasive cervical cancer, according to the WHO histological criteria.

Loop electrosurgical excision procedure (LEEP) was performed for treatment of CIN2 or worse (CIN2+). After the LEEP operation, re-examinations of HPV genotyping and cytology co-testing were performed every 3 months of follow-up, and colposcopy examination was performed for patients with abnormal follow-up results. Data for HPV genotyping, cytology tests and histological diagnoses were obtained from our Taizhou Area HPV Study [8] with longitudinal follow-up for at least 5 years.

### Statistical analysis

SPSS 16.0 statistical software (SPSS Inc., Chicago, IL) was used for this study. The association of cervical carcinogenesis risk with HPV16 variants was analysed using the chi-square test or Fisher’s exact test. Odds ratios (ORs) and relative 95% confidence intervals (95% CI) were calculated. All statistical tests were two-sided. *P* values < 0.05 were accepted as statistically significant.

## Results

### Characteristics of the study population

**Fig. 1:**
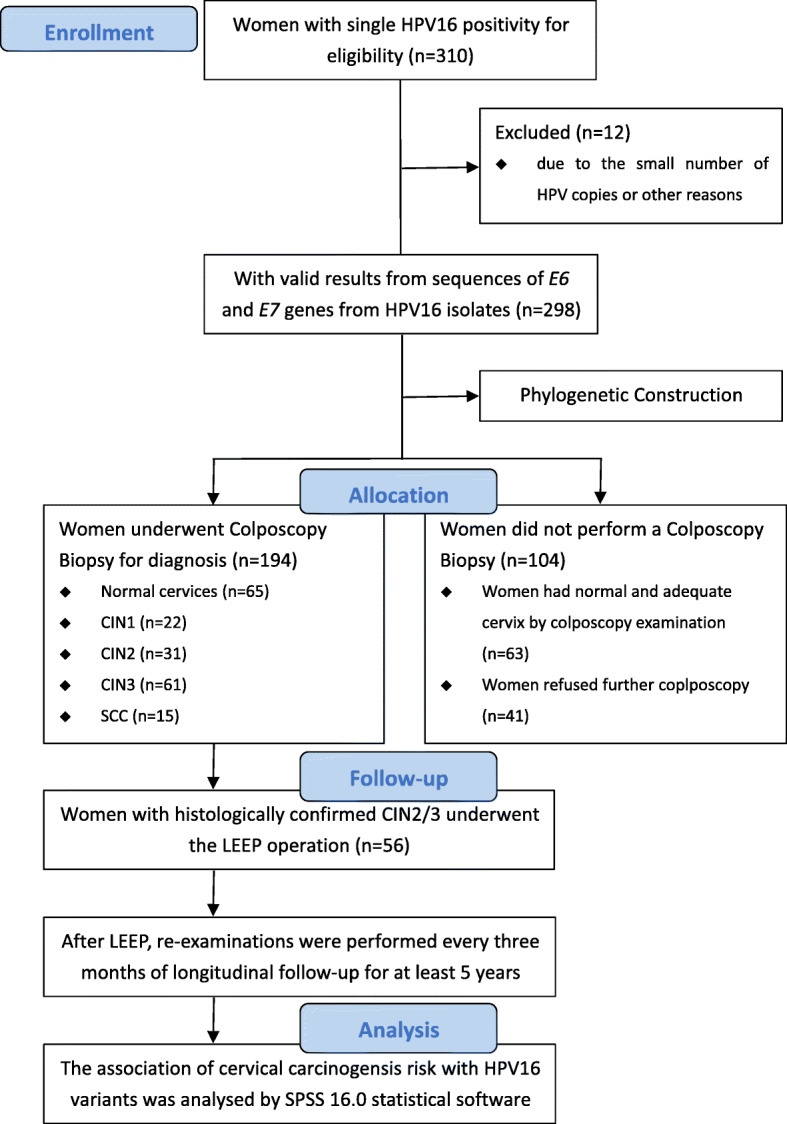
Flow diagram of the present study

Between December 2012 and December 2014, single HPV16 positivity was detected in 310 women (median age 41.6 years; range 19–77) who were selected for this study. The flow diagram of the present study is shown Fig. 1. A total of 298 (96.1%) sequences of the *E6* and *E7* genes from HPV16 isolates were obtained. Twelve (3.9%) sequences were excluded due to the small number of HPV copies. Among 298 HPV16-infected women, 63 (21.1%) had normal and adequate cervix by colposcopy examination, so no further biopsy diagnosis was performed. 41 (13.8%) women refused further colposcopy examination. The remaining 194 (65.1%) underwent colposcopy biopsy for diagnosis which were further used for risk association analysis, and 65 were diagnosed with normal cervices after biopsy, 22 with CIN1, 31 with CIN2, 61 with CIN3, and 15 with squamous cell carcinoma (SCC). Characteristics of the study population categorized by HPV16 (sub)lineage are shown in Table 1.

**Table 1:** Distribution of HPV16 (sub)lineages according to cervical disease status (*n* = 194)

Ages	NILM	CIN1	CIN2	CIN3	SCC	Total
	42.2 ± 9.8	42.7 ± 8.5	39.6 ± 9.9	43.6 ± 6.7	55.3 ± 11.7	
A1	4	2	1	2	1	**10**
A2	8	3	5	5	2	**23**
A3	5	5	3	6	0	**19**
A4^*^	40	11	19	45	10	**125**
A5	7	0	1	2	2	**12**
non-A	1	1	2	1	0	**5**
**Total**	**65**	**22**	**31**	**61**	**15**	**194**

*NILM* negative for intraepithelial lesion or malignancy, *CIN* cervical intraepithelial neoplasia, *SCC* squamous cell carcinoma

^*^The A4 (Asian) variants had a higher risk of CIN2+ than the A1–3 (European) variants when compared to CIN1 (OR = 2.69, 95% CI = 1.04 to 6.97, *P* < 0.05)

### Variations in the E6 and E7 genes

**Fig. 2:**
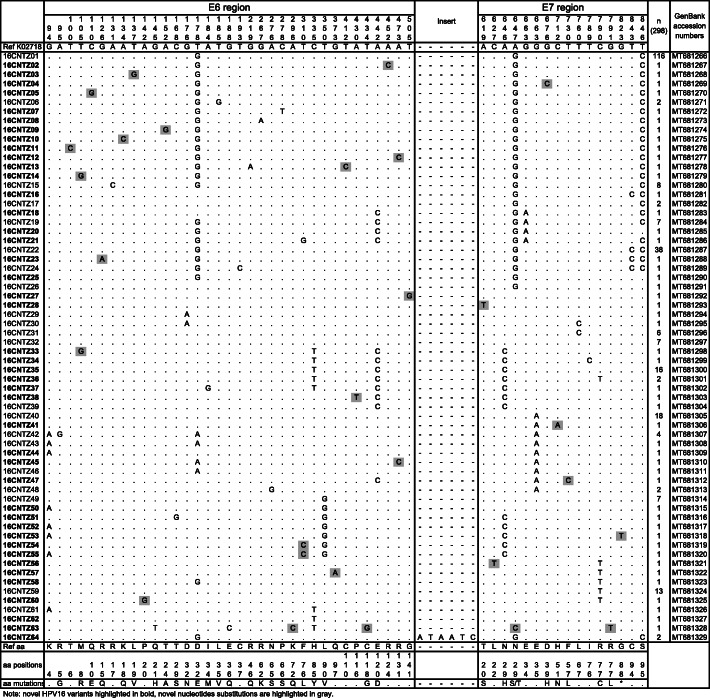
Genetic variability of HPV16 *E6* and *E7* nucleotide sequences in Taizhou area, Southeast China. Numbering refers to the first nucleotide of the HPV16 prototype reference sequence (GenBank: K02718). Each row indicates the isolate identification and the nucleotide sequence alignment compared to the reference. Novel HPV16 variants are highlighted in bold and novel nucleotide substitutions are highlights in gray

Compared with the prototype HPV16 reference sequence K02718, 97.7% (291/298) of the HPV16 isolates showed nucleotide variation in our study. A summary of nucleotide and amino acid sequence variation throughout the E6 and E7 fragments is shown Fig. 2. We obtained 64 distinct variation patterns denoted as 16CNTZ01-16CNTZ64, which were published with the GenBank accession codes MT681266 to MT681329. In this study, 43 (67.2%, 43/64) novel HPV16 variants were detected, which are highlighted in bold in Fig. 2. It is worth noting that the insertion of ATAATC between nt561 and nt562 was first detected in the 16CNTZ64 variant, which accounted for 0.7% (2/298) of HPV16 isolates.

In the *E6-E7* sequences, a total of 54 single nucleotide substitutions were identified, with 36 (66.7%) non-synonymous substitutions and 24 (44.4%) novel substitutions. The sequence variability in the *E6* gene was higher than that of the *E7* gene (Fig. 2). The three most prevalent nucleotide substitutions were T178G (D32E) (191/298, 64.1%) in the *E6* gene and A647G (N29S) (195/298, 65.4%) and T846C (192/298, 64.4%) in the *E7* gene, which are specific to the A4 (Asian) sublineage. Another nucleotide substitution was also found in nucleotide site 178, T178A (D32E), with the same amino acid change as T178G but belonging to the A3 (European) sublineage. Notably, C335T (H85Y) was previously found to be specific to B/C/D lineages, and A646C (N29H) belongs to the A2 sublineage. However, both C335T (H85Y) and A646C (N29H) non-synonymous substitutions appeared in 7% (21/298) of HPV16 isolates at the same time in this TZHPV study.

To the best of our knowledge, the base substitutions of T100C, T105G(M8R), C110G(Q10E), G126A(R15Q), A134C(K18Q), T137G(L19V), A142G, A152G(T24A), A296C(K72Q), T310G(F76L), G373A, T412C, A430T, T434G(C118G), A452C, A473C, and T505G in *E6* and A619T(T20S), C627T, A647C(N29T), G676C(D39H), T730C(F57L), G791T(R77L), and G823T(G88*) in *E7* have never been reported in previous studies.

### Phylogenetic construction

**Fig. 3:**
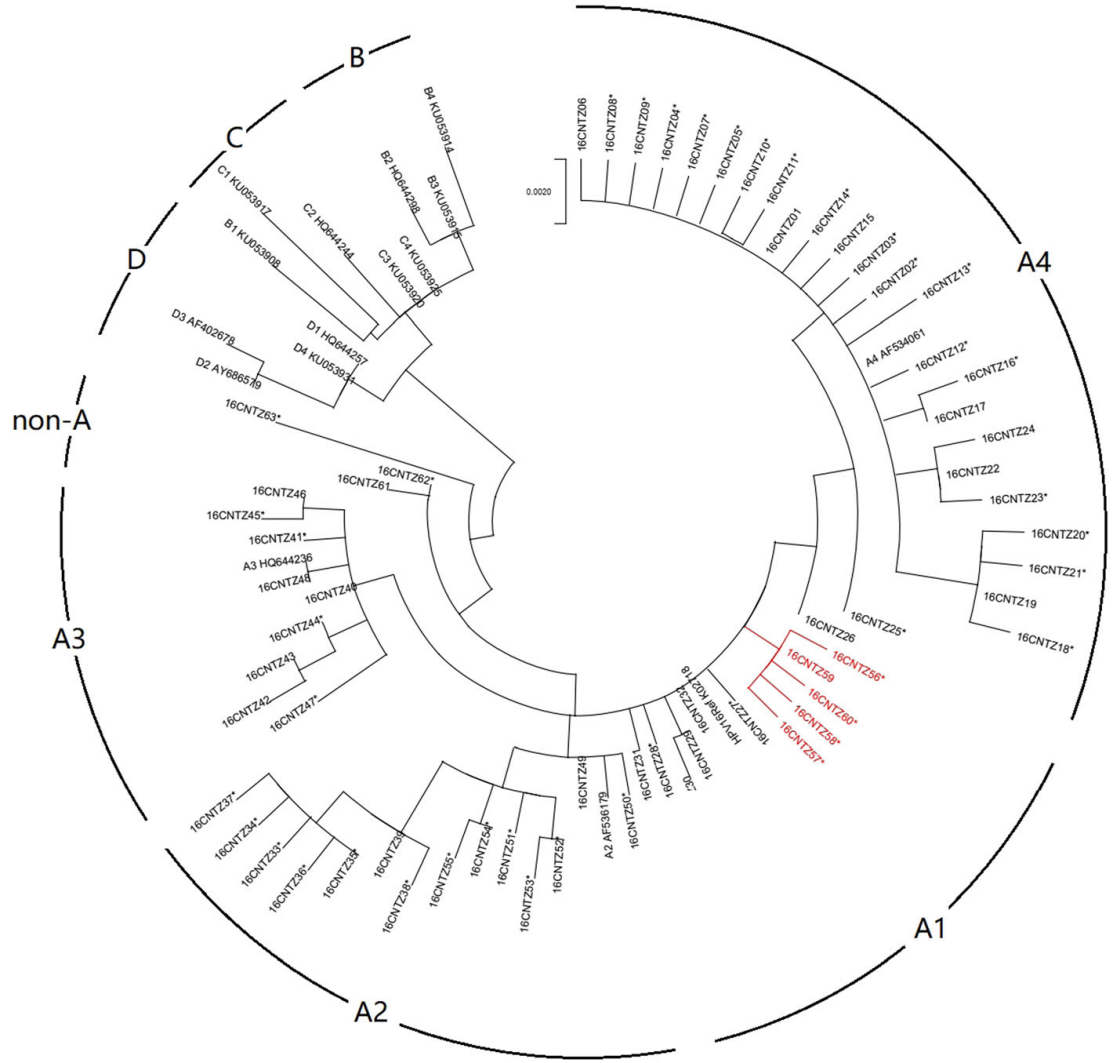
Phylogenetic tree of the HPV16 variants. Maximum-likelihood analysis (with MEGA X) of E6/E7 nucleotide sequences was inferred from 64 obtained HPV16 variants and 16 reference sequences. Numbers below branches indicate bootstrap values

The maximum-likelihood phylogenetic tree based on the HPV16 *E6-E7* sequences was inferred from 64 obtained HPV16 variants and 16 reference sequences (Fig. 3). According to the phylogenetic tree, 93.8% (60/64) of HPV16 variants belong to lineage A. In our study, the most predominant HPV16 variants belong to sublineage A4 (64.8%, 193/298), followed by sublineages A2 (12.1%, 36/298), A1 (11.4%, 34/298), and A3 (10.0%, 30/298). Notably, the 16CNTZ61, 16CNTZ62, 16CNTZ63 and 16CNTZ64 variants representing 5 samples (1.7%) belonging to non-A variant lineages were also identified in the Taizhou area.

16CNTZ01 is the most common variant (38.9%, 116/298) in our population, followed by 16CNTZ22 (12.8%, 38/298) and 16CNTZ40 (6.0%, 18/298). The novel variant 16CNTZ35 was the most common variant (44.4%, 16/36) in the A2 sublineage. A subset of the A2 sublineage appeared to be uniquely defined by the C335T (H85Y) and A442C (E120D) in *E6* and A646C (N29H) in *E7,* and a subset of the A1 sublineage was uniquely defined by the C790T (R77C) in the *E7* gene.

### Risk association with cervical carcinogenesis

Among women with histologically confirmed abnormal cervixes, the A4 (Asian) variants had a higher risk of CIN2+ than the A1–3 (European) variants (OR = 2.69, 95% CI = 1.04 to 6.97, *P* < 0.05) (Table 1). It was clear that A4 variants had stronger oncogenicity than A1–3 variants in the Taizhou population. Moreover, our data showed that the oncogenicity of HPV16 *E6* T178G (D32E) and *E7* A647G (N29S) variation was associated with an increased risk of CIN2+ (OR = 2.24 ~ 2.45) (Table 2).

**Table 2:** Distribution of major nucleotide variation in HPV16 E6 and E7 genes based on cervical disease status

Gene	Nucleotide variation	Amino Acid variation	Women with cervical disease status					
			NILM ***n*** = 65	CIN1 ***n*** = 22	CIN2 ***n*** = 31	CIN3 ***n*** = 61	SCC ***n*** = 15	Total ***n*** = 194
**synonymous substitutions**								
E6	A95G	R5G	0	1	1	0	0	**2**
	T105G	M8R	0	0	1	1	0	**2**
	C110G	Q10E	1	0	0	0	0	**1**
	A134C	K18Q	0	0	0	0	1	**1**
	T137G	L19V	0	0	0	1	0	**1**
	G145T	Q21H	0	1	0	0	0	**1**
	A152G	T24A	0	0	0	1	0	**1**
	C168G	T29S	0	0	1	0	0	**1**
	T178G	D32E	38	11	18	46	10	**123**
	T178A	D32E	0	2	1	2	0	**5**
	T185G	L35V	0	0	0	1	0	**1**
	G188C	E36Q	0	1	0	0	0	**1**
	A296C	K72Q	0	1	0	0	0	**1**
	T310C	F76L	0	1	1	0	0	**2**
	C335T	H85Y	4	2	3	4	2	**15**
	T350G	L90V	3	2	3	1	0	**9**
	T434G	C118G	0	1	0	0	0	**1**
	A442C	E120D	7	2	2	8	2	**21**
E7	A646C	N29F	5	3	4	5	2	**19**
	A647G	N29S	40	11	20	46	10	**127**
	A647C	N29T	0	1	0	0	0	**1**
	C712A	H51N	0	0	0	1	0	**1**
	T730C	F57L	0	0	0	1	0	**1**
	C790T	R77C	7	0	2	3	2	**14**
	G791T	R77L	0	1	0	0	0	**1**
	G823T	G88*	0	1	0	0	0	**1**
**non-synonymous substitutions**								
E7	G663A	–	2	1	0	3	0	**6**
	G666A	–	5	5	3	6	0	**19**
	T843C	–	4	1	5	10	1	**21**
	T846C	–	40	11	19	44	10	**124**
	others	–	9	6	5	8	1	**29**
	**Total**		**165**	**66**	**89**	**192**	**41**	**553**

*NILM* negative for intraepithelial lesion or malignancy, *CIN* cervical intraepithelial neoplasia, *SCC* squamous cell carcinoma

The symbol *reflected terminal codon, the end of the protein sequence

At follow-up, 56 women with histologically confirmed CIN2/3 underwent the LEEP operation, among whom, 10 women were diagnosed as the residual/ recurrent disease during the follow-up visit. Seven women with positive margin: 3 women with HPV16 positive and cytology normal, 1 women with HPV16 positive and cytology abnormal, 2 women with HPV negative and cytology abnormal, and 1 women with histologically confirmed CIN3 at the 3rd month follow-up visit. Five women had recurrent CIN2/3 (rCIN2/3) within 5 years post-treatment, of which 3 women with clean margins: 1 had 16CNTZ01(A4), 1 had 16CNTZ25(A4), and 1 had 16CNTZ51(A2). At follow-up, 65 women with histologically confirmed normal cervix, among whom, 6 women were progressed to CIN (1CIN1, 3CIN2 and 2CIN3) within 5 years: 3 had 16CNTZ01(A4), 1 had 16CNTZ22(A4), 1 had 16CNTZ59(A5), and 1 had 16CNTZ61(non-A). Additionally, the substitution G823T at E7 leading to a premature stop codon occurred in an isolated CIN1 sample (16CNTZ53/A2), and follow-up for 3 years showed a normal cytology test.

## Discussion

China is one of the top contributors to the global burden of cervical cancer, of which HPV16 causes 70% of all cervical cancer worldwide, and some HPV16 variants are more oncogenic than others [13, 21]. It has been clear that the distribution of HPV16 (sub)lineages depends on geographical origin and ethnicity, combined with more recent migration patterns (especially migration from Europe and Africa to the Americas) [22, 23]. Globally, the data showed that variants in the HPV16 sublineages A1–3 were the most widespread in Europe, lineage D in Central-South America, and sublineage A4 in Asia, and lineages B and C variants were mostly restricted to Africa [24]. Several epidemiological studies have suggested that HPV16 non-European variants are associated with the persistence of HPV infection and its progression to cervical cancer, especially sublineages A4, C, D2, and D3 [11, 14, 16, 17, 24, 25].

In this study, we aimed to shed light on the HPV16 variants currently circulating in the Taizhou area, China. We obtained 298 complete sequences of the *E6* and *E7* genes from HPV16 isolates. Most of the HPV16 variants belong to the A lineage (98.3%), of which the A4 (Asian) sublineage was dominant (64.8%). The results of our study are in agreement with previous data in Central (60.3%) and South China (65.5%) [26–29]. In China, HPV16 variants in the A1–3 (European) sublineages are common in Xinjiang, which lies in Northwest China [30, 31]. Our results indicated that the A4 (Asian) variants had an increased risk for CIN2+ compared to the A1–3 (European) variants (*P* < 0.05), which is in accordance with the previous findings reported in a pooled worldwide analysis (adjusted by country) [14]. Thus, the higher contribution of HPV16 to cervical cancer in China may be due to the higher oncogenicity of sublineage A4 (Asian) variants [25]. To our knowledge, the number of HPV16 isolates analysed in this study represents the largest reported collection sampled in Southeast China.

As the HPV16 oncoproteins play an important role in the development of cervical cancer, nucleotide variations in the HPV16 *E6* and *E7* genes could also be associated with the progression of cervical carcinogenesis. Here, we identified 54 single nucleotide substitutions in HPV16 isolates in the Taizhou population, including 37 in the *E6* gene and 17 in the *E7* gene (Fig. 2). In particular, we found that the three most common variations in HPV16 isolates were the T178G (D32E) in E6 (64.1%) and A647G (N29S) and T846C in E7 (65.4 and 64.4%, respectively), and these three nucleotide substitutions are apparently linked because of their simultaneous occurrence in 96.4% of A4 (Asian) variants in this study. It has been reported that the prevalence of both non-synonymous substitutions D32E and N29S is much higher in Asia (65.5% in China, 68.0% in Korea, 44.2% in Japan and 73.9% in Thailand) than in Europe (2%), North America (3%), and Africa (0%) [25, 32–35]. Our results showed that the oncogenicity of E6 D32E and E7 N29S variations was associated with the development of cervical cancer. It has been confirmed that the non-synonymous substitution A647G(N29S) may block the physiological function of Rb, thereby maintaining long-term infection of HPV and increasing the likelihood of persistent viral infection and cervical cancer progression [31, 36, 37]. Therefore, these data may help to explain the higher cervical cancer burden observed in China.

T350G (L90V) has been shown to be associated with the progression of cervical lesions [38–40]. Many studies have reported that 350 T/G polymorphism is a common variation located in the *E6* oncogene, but it has significant heterogeneity by the world region [41]. In Europe/Central Asia and East Asia, cervical cancer risk was significantly associated with the 350 T. However, the opposite was true in South Asia and South/Central America [40, 41]. In our study, we did not find that HPV16 *E6* T350G (L90V) was associated with cervical cancer risk, which is in good agreement with previous data in China [28]. Notably, 350 T/G polymorphism has been shown to influence the cervical cancer risk of European lineages, and also occurs in non-European lineages [40]. Additionally, HPV16 E6 H85Y and E120D belong to the A2 sublineage in this study but are not associated with cervical cancer. Therefore, the carcinogenicity of 350 T vs 350G, 85H vs 85 V, 120E vs 120D might be population-dependent. E6 variations might contribute to oncogenesis by disrupting p53 degradation by affecting the interaction between E6 and p53/E6AP.

Our follow-up data showed that 56 women with HPV16-positive CIN2/3 were treated by the LEEP operation, and 10 women (17.9%) were diagnosed as the residual/ recurrent disease during the follow-up visit. Among 5 women with rCIN2/3, 80% (4/5) of HPV16 isolates belong to the A4(Asian) sublineage. The reasons for the residual/ recurrent after the LEEP operation might be incomplete excision of baseline CIN2/3, high-risk HPV DNA persistence, or multifocal disease. Our follow-up data also showed that 65 women with histologically confirmed normal cervix, among whom, 6 women (9.2%) were progressed to CIN grade. 66.7%(4/6) of HPV16 isolates belong to the A4(Asian) sublineage. The persistence HPV16 DNA is the root cause of the recurrent disease for the women treated for the CIN2/3, and the same HPV16 variants were found in both lesions [42]. Therefore, our follow-up data showed that 24.7% (48/194) women with persistent infection of HPV16 genotype, which may involves a persistent infection with the same variant.

The largest limitation of our study was the time of follow-up. Since the time between HPV16 infections and CIN2/3 lesions was calculated to be 5–15 years post-infection, our longitudinal follow-up for HPV genotyping, cytology tests and histological diagnoses may affected the outcome in the present study.

## Conclusions

In summary, the present study reported genetic variants in the HPV16 *E6* and *E7* genes in the Taizhou area, Southeast China, and provided evidence for their involvement in the increased risk of cervical carcinogenesis. Data about different HPV16 variants in the population of specific regions have significance in uncovering the carcinogenic mechanism of HPV16 and in developing preventive and therapeutic vaccines against HPV.

## Supplementary Information

**Additional file 1.** Clinical data for HPV16 study in Taizhou area, China.

## Data Availability

All data generated during this study are included in this published article. The supplementary materials included the nucleotide variations of the *E6* and *E7* genes from HPV16 isolates and the follow-up data of patients. In addtion, these sequences have been released to GenBank database with the accession codes of MT681266 to MT681329. The links are https://www.ncbi.nlm.nih.gov/nuccore/MT681266 ~ https://www.ncbi.nlm.nih.gov/nuccore/MT681329.

## Abbreviations

CIN: Cervical intraepithelial neoplasia
HPV: Human papillomavirus
LCR: Long control region
LEEP: Loop electrosurgical excision procedure
ORF: Open reading frames
PCR: Polymerase chain reaction
